# As Well as We Know

**Published:** 1879-07

**Authors:** J. J. Keyes


### AS WELL AS WE KNOW.

J. J. KEYES.

It is a saying more or less common, that "if we would do as well as we know, we would do well enough." The saying is hardly correct; because the highest knowledge is essential to the best doing; and only the best doing is "well enough." But if it be said: "If we would do as well as we know, we would do vastly better than we do," the saying would be above criticism; for no living man can honestly say: "I am doing, in all things, to the very best of my knowledge." Every man is violating, to some extent, the rules and principles of life and action with which he has become acquainted, and which he knows to be wise and safe. No man's life is altogether shaped as his knowledge would enable him to shape it, were he always guided and governed by his best judgment.

This remark is true concerning the *laws of health*.

True, very few people, comparatively speaking, are extensively informed in regard to these laws. They know but little of the constitution of their own bodies. Physiology is, to them, a sealed book. They hardly know whether the liver is located in the right leg or over the left shoulder; whether the heart has to do with the blood or with the respiration; whether the bronchial tubes have connection with the lungs or with the intestines. Concerning the chemical elements that enter into the composition of the human body, and concerning the kinds of food required for their supply and sustentation, they are equally ignorant. The specific offices of bread, meat, fish, eggs, sugar, and of various fruits and vegetables, as articles of diet, are not understood and are seldom thought of. The precise effect upon nerve and tissue, of alcoholic stimulants, and the real dangers attending their use, are matters that most people who use them do not stop to consider and have never made a study. In short, notwithstanding the attention now given to physiology and hygiene in nearly all of our schools, notwithstanding the information imparted on this subject by the newspapers, magazines and medical journals, notwithstanding the attempts of our educated physicians to inculcate useful lessons in health among the families which enjoy their profes-sessional services—the fact yet remains that hygiene, as a science, is very imperfectly understood by the masses of the people. And it must be said that the knowledge, even of the best informed on this subject, is, as yet, largely relative, empirical and imperfect. Hygiene, as a science, is only in its infancy. Dietetics, at its present stage, can hardly be called a science at all. What shall we eat, and how shall we cook it, are yet questions that demand further solution. The wonder, in view of the general ignorance, is that people are as well as they are, and live as long as they do. The human body seems capable of enduring a vast amount of neglect and abuse. Men live in spite of their ignorance—in spite of their attempts to destroy themselves.

And yet, making all due allowance for their ignorance, very few people *do* as well as they *know*. They have learned many things by experience, observation, and study, that are valuable, and might be made useful; but they do not practice what they have learned. People know that when their blood is heated and their bodies are in a perspiration, they should not sit in a draft of cool air; or go into a cold cellar, or into an ice house to reduce the temperature that oppresses them. And yet people are doing these very things every day this summer; and, every day they are taking cold as the result, and, in many cases, are planting the seeds of consumption and death.

There are people who know they cannot eat certain vegetables—cucumbers for instance—with impunity; but they *like* cucumbers, and will eat them, knowing that, in all probability, within six hours afterward, they will be bent double and turned inside out with cholera morbus or diarrhœa. The boys who eat green apples, know the same things, and experience the same results.

There are ladies and ladies, young, middle-aged and aged, who have been taught by physicians, and whose common sense teaches them that art cannot add to the real grace and beauty of the "human form divine;" who know that a small, slender, taper waist is not nature's product, and that undue compression at that point interferes with the normal, easy action of all the vital organs, and endangers health and life; and yet they will persist in wearing the abominable corset.

I do not claim the possibility of perfect health at all times, in all places, and for all people, even when all known laws of health are understood and faithfully obeyed. Atmospheric changes, unfavorable to health, may occur at any time. Malarial influences may exist—may surround us—may poison our blood and lead to fever and death—whose existence we had no reason to suspect. Certain epidemics sweep away the intelligent and the uninformed, the careful and the imprudent, the clean and the unclean alike, in utter disregard of all sanitary precautions and regulations.

But, these exceptional causes aside, we should, unquestionably, escape many of the ills that flesh is heir to, if we gave a decent, consistent observance to the rules of health with which we have become familiar. Let every man set about doing as well as he knows, and we shall soon have fewer sick and complaining people.

What a blessing it would be, also, if people would only do as well as they know, as regards their conduct in life! The principles of righteousness are plain, simple, and easy of comprehension. And they are generally understood. Man intuitively apprehends them. Stanley shows that even the natives of Central Africa are not without notions of rightness. The North American Indian, however treacherous he may be, knows when he is fairly and rightly treated by the agents of our government, and is quick to resent his wrongs. The Golden Rule is older than Jesus; as old as Osiris, Zoroaster, Buddha, Confucious; it is as old as human intelligence, the human conscience, the human race. The human mind has native perceptions of things just, right and good. These may be and often are dimmed and blunted in men who persistently violate them, and in races which have not yet emerged from barbarism. But these perceptions are not any where totally extinct; and in all civilized countries, certtain definite standards of right acting are found ; questions of various kinds are all the time being discussed with reference to their ethical quality and bearing; so that the sense of rightness in the human mind, is in no danger of dying out. All religions involve this principle, and derive their very largest and best significance from this fact. So that while many questions that engage our attention, are referred to our pride, vanity, or selfish greed, most questions are, in some sense, referred to our conscience, our sense of rightness, fairness, justice. It is the office of religion to keep this sense alive and active.

Now, I believe that this sense is sufficiently developed to produce right conduct, and to prevent damage by one man to the interests of another, provided every man should *do* as well as he *knows*. At all events, I believe this of all the readers of the BISTOURY. The merchant does not need that the preacher should stand by his side and tell him it is wrong for him to defraud his customer. The employer does not require to be told that he should not oppress his employe, by compelling him to overwork for under-pay.

The speculator need not be informed that he sins against his fellowmen, when he makes a "corner" in some great staple, and demands a price so extortionate that only the rich and well-to-do can buy. The men and women are very few who need to be told that such and such acts, such and such tempers of mind, such and such courses of conduct are a wrong to themselves, or a damage to their neighbor. Mrs. Tellall knows very well that she has no business to retail to all her acquaintances the secret confided to her by some trusting friend. Mrs. Surmise knows it is wrong and contemptible to judge from appearances, and then to speak evil of those who are probably a thousand times better than herself. Mrs. Meddler knows that to get people by the ears, is about the smallest, lowest, meanest business she could be engaged in. The backbiter, defamer, and slanderer, whether man or woman, knows better than to act in these despicable characters. His innate sense of right tells him it is wrong. His innate sense of nobility tells him it is mean. Society suffers mainly from the wrong doing of people who know better. Many existing evils would disappear, and many wrongs would be righted, if all would do as well as they know. As in the matter of health, so in the matter of conduct, it is not more knowledge that is needed, so much as it is an honest determination and attempt to use to advantage the knowledge already possessed. The people's health would improve; the people's character and conduct would develop into surprising purity, righteousness and beauty; and society would speedily reach comparative harmony and perfection, if the people, one and all, would act up to their highest intelligence and their highest sense of rightness. For "if we would do as well as we know, we would do vastly better than we do."

